# Bibliometric analysis of publications on *Campylobacter*: (2000–2015)

**DOI:** 10.1186/s41043-016-0076-7

**Published:** 2016-11-29

**Authors:** Waleed M. Sweileh, Samah W. Al-Jabi, Ansam F. Sawalha, Adham S. AbuTaha, Sa’ed H. Zyoud

**Affiliations:** 1 Department of Physiology, Pharmacology and Toxicology, College of Medicine and Health Sciences, An-Najah National University, Nablus, Palestine; 2 Department of Clinical and Community Pharmacy, College of Medicine and Health Sciences, An-Najah National University, Nablus, Palestine

**Keywords:** *Campylobacter*, Bibliometrics

## Abstract

**Background:**

*Campylobacter* species are widespread zoonotic pathogens. *Campylobacter jejuni* causes a form of gastroenteritis called campylobacteriosis. *Campylobacter* drug resistance is considered a serious threat. In order to better understand national and international research output on *Campylobacter*, we conducted this bibliometric overview of publications on *Campylobacter*. This study can be used to assess extent of interaction and response of researchers, food regulators, and health policy makers to global burden of campylobacateriosis.

**Methods:**

Scopus database was used to retrieve publications with the following keywords (*Campylobacter/campylobacteriosis*, *C. jejuni*, *C. coli*). The study period was set from 2000 to 2015. All types of journal documents, excluding errata, were considered. Bibliometric indicators such as annual growth of publications, country contribution, international collaboration, and citation analysis were presented. The quality of retrieved data was indirectly assessed by Hirsch index and impact factor of journals.

**Results:**

A total of 5522 documents were retrieved with median (Q1–Q3) citations of 9 (2–23) and *h*-index of 113. Annual number of publications showed a fluctuating increase. The core leading journals were *Applied and Environmental Microbiology* journal and *Journal of Food Protection* with 246 (4.46%) publications for each. The USA (1309; 23.6%) was the most productive country while Danmarks Tekniske Universitet (150; 2.7%) was the most productive institution. Half of the top ten productive countries were European. France had the lowest percentage (33.5%) of articles with international collaboration while Netherlands (57.7%) had the highest percentage of articles with international collaboration. Approximately half (50.1%) of retrieved articles were published in journals under the subject area of “immunology/microbiology”. Main themes in highly cited articles were molecular biology/genetics and public health burden of campylobacteriosis. There were 728 (13.1%) articles on campylobacter-related drug resistance, and the top cited articles focused mainly on increasing resistance to quinolones and fluoroquinolones.

**Conclusions:**

There was a clear increase in number of publications on *Campylobacter.* Rational use of antimicrobials in humans, poultry, and animals is highly recommended. International collaboration is highly required particularly in implementing new diagnostic screening technologies to minimize global health burden of *Campylobacter* and ensure food safety.

## Background

*Campylobacter* species are zoonotic pathogens [1]. Many animals and birds, particularly broiler chickens, carry *Campylobacter* asymptomatically and shed the microorganism in their feces [2]. *Campylobacter jejuni* is the most important species of *Campylobacter*. The pathogen is transmitted to people mainly through eating undercooked poultry meat or from contaminated food or animal products [3]. *C. jejuni* has been implicated in a form of gastroenteritis called campylobacteriosis [4]. People with campylobacteriosis will have diarrhea, cramping, and fever within few days of exposure to *Campylobacter* [5–7]. Asymptomatic *Campylobacter* infections are usually common and endemic in developing regions like Middle East, Africa, and South Asia, with children being most affected [8–10]. However, asymptomatic *Campylobacter* infections are uncommon in developed regions [11].

The exact incidence of campylobacteriosis at the global and national levels is poorly known. However, the past decade has witnessed an increase in global incidence of campylobacteriosis [7, 12]. A survey study on foodborne illnesses in the USA indicated that 9% of these illnesses were caused by *Campylobacter* compared to 58% for norovirus [13]. In the USA, *Campylobacter* is the most common bacterial cause of diarrheal illness [14]. It is also estimated that *Campylobacter* affects over one million people every year causing death in 76 patients every year in the USA [15]. In the UK, it has been reported that campylobacter affects an estimated half a million people annually and kills approximately 100 [16]. In the UK, the reported incidence of campylobacteriosis was estimated to be 9.3 per 1000 person-years while that in Netherlands was 5.8 per 1000 person-years [17–19].

In response to worldwide concern of foodborne gastroenteritis caused by *C. jejuni*, World Health Organization (WHO) is developing policies that will further promote the safety of entire food chain [20]. Campylobacteriosis is usually a self-limiting disease. However, in certain cases, antibiotics such as macrolides or flouroquinolones might be needed. Unfortunately, worldwide reports of drug-resistant *Campylobacter* in humans, poultry, and food have been published which threatens our goal of treating millions of people around the world [21–26]. The CDC considers *Campylobacter* drug resistance as a serious threat [27].

There are several published bibliometric studies on specific pathogenic bacteria or gastrointestinal diseases, or zoonosis in general [28–33]. However, none was carried out about *Campylobacter* and its related drug resistance profile. Therefore, we carried out this bibliometric analysis to assess worldwide research productivity on *Campylobacter*. In specific, the following bibliometric indicators will be presented in this study: annual growth of publications, citation analysis, country and institutional contribution, highly cited articles, international collaboration, and journals most active in publishing research on *Campylobacter*.

This study is in line with the WHO recommendations in its latest report on campylobacteriosis in which the panel of consultation experts recommended several actions to be taken to better understand and control *Campylobacter* [34]. Furthermore, diarrheal diseases are considered to have major global burden, and research in this area is needed [35]. This study will be important for researchers, clinicians, and health policy makers in order to adopt stringent policies regarding veterinary practices, food processing, antibiotic use, and preventive measures to control campylobacteriosis. Furthermore, this bibliometric analysis can assess areas of national and international research strengths, highlight knowledge and evidence gaps, provide a dataset of institutional and individual expertise which explore different disciplines covering campylobacter-related health research.

## Methods

For the purpose of this study, the authors chose Scopus database (https://www.scopus.com) to retrieve all possible documents related to *Campylobacter*. Of course, the choice of Scopus was based on the understanding of the authors that it is the largest and most comprehensive database [36]. Furthermore, Scopus has been previously used in many published bibliometric studies [37–42].

The search strategy used in this study consisted of several steps. The first step was finding the correct and comprehensive keywords. For the purpose of this study, we use the following keywords: Campylobacter* or “C. jejuni” or “C. coli”. The asterisk was used in search query to retrieve documents with keywords such as *Campylobacter* or campylobacteriosis. The keywords “C. jejuni” and “C. coli” were used in search query because some publications might include the abbreviation name instead of full name, and therefore, these terms were used in search query. Quotation marks were used to increase accuracy of search query. Keywords were used in title search rather than title/abstract search. The title search will yield minimum false positive documents, and therefore, it is an accurate approach. However, the title/ abstract search will yield many false positive results in which the main focus is not on *Campylobacter* per se [43–51]. It is true that “title search” might lead to loss of some documents (false negative), but the error (false positive) obtained by “abstract search” will be greater. In this study, the authors tried the title/abstract search and title search scenarios and found that there were 3306 document differences between the two scenarios. Manual review by authors of top 100 cited articles of these 3306 documents showed that *Campylobacter* keyword was mentioned in these articles as a marginal keyword rather than an essential part of the article itself. Based on this, the authors decided to go with the title search rather than the title/ abstract search.

**Fig. 1:**
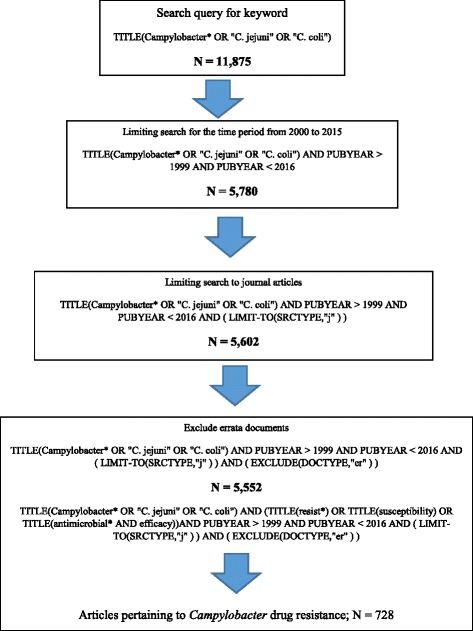
A scheme for the method used to retrieve campylobacter-related articles in Scopus

The second step in the search strategy was to limit the search query to the required specifications. In this study, the search query was limited to study period between years 2000 and 2015. This is easily done because Scopus allows researchers to specify the time period for the search query. Scopus also lists the type of retrieved documents, e.g., journal articles or books. In this study, only journal articles were included in the study while non-journal articles such as books and book chapters were excluded. Finally, some journal articles were actually just corrections of mistakes in previously published articles. Such corrections (errata documents) were excluded because they do not represent a publication. Figure 1 shows a scheme of the steps followed to retrieve data pertaining to this study. In each step, the total number of documents retrieved was mentioned.

Scopus has a function called languages. In this function, the frequency of articles written in English or any other languages is listed. No articles were excluded based on language. Based on Scopus policy, all published articles indexed in Scopus must have an English abstract. Therefore, even articles with non-English language such as Chinese do have an English title and abstract that facilitates content analysis.

The third step after obtaining the required documents was to analyze the data to provide bibliometric indicators. One of these bibliometric indicators is national research productivity and extent of international collaboration. Scopus provides automatic country analysis for any retrieved data. Scopus has a side bar with functions that can help researchers analyze data and obtain the required indictors. One of these functions is country affiliation. In Scopus, publications can be divided into two types: (1) single country publications (SCP) which represent inter-country publication because the authors of the publication belong to different countries. Other bibliometric indicators to be obtained from retrieved data include the most active institutions, journals, and authors. Furthermore, retrieved data can be categorized based on subject areas. This function is available in Scopus side bar in which documents are categorized based on the scope of the journal publishing that document. For example, subject area “medicine” which includes all articles published in journals within the scope of medicine. The same applies to subject area “Biochemistry” and other subject areas. All these bibliometric indicators can be obtained from Scopus directly by using the bar side functions of Scopus which include functions such as intuition, author, and journals.

Strength and impact of publications which represents quality is difficult to measure in a direct way. However, several indicators could be used to evaluate the strength and quality of publications in an indirect way. Such indirect quality indicators include the total number of citations received, average number of citations per article, Hirsch index (*h*-index), percentage of highly cited articles, and impact factor (IF) of journals publishing the documents of interest. *h*-index was originally developed to assess and rank researchers [52]. However, *h*-index can be used to rank countries and academic institutions [52]. In this study, *h*-index was obtained directly from Scopus database while IF was obtained from the Journal Citation Reports 2015 published by Thompson Reuters [53].

The standard competition ranking (SCR) was used for ranking purposes of most productive (active) countries, institutions, journals, and authors. Graphs were created using the Statistical Package for Social Sciences software (SPSS for windows, 21) while tables were created using Microsoft Excel. Ethical approval of this study was not required by the Institutional Review Board since no human subjects or data were involved. All data analyses were carried out on October 25th, 2016. The number of publications retrieved in this study can be retrieved by other researchers with high reproducibility using the search query shown in Fig. 1. Furthermore, the excel sheet of retrieved articles is attached for those who are interested (annex 1).

## Results

### General information

The search query yielded 5552 journal articles (Fig. 1). A total of 5160 (92.9%) articles were written in English while the remaining articles were written in non-English, such as Spanish (72, 1.3%), German (58, 1.0%), Polish (52, 0.9%), Chinese (51, 0.9%), and French (41, 0.7%). A total of 4889 (88.0%) articles were research articles while the remaining were review articles (294, 5.3%), letters (164, 3.0%), conference papers (71, 1.3%), notes (58, 1.0%), short survey articles (35, 0.6%), articles in press (27, 0.5%), and editorials (14, 0.3%).

### Growth of publications and citation analysis

The annual number of publications showed a fluctuating rise with time and reached a maximum of 448 articles in 2014. There was a twofold increase in the number of publications during the study period. The total number of citations for retrieved articles was 110,160, an average ± standard deviation of 19.8 ± 38.5 citations per article. The median (interquartile range) of the number of citations received was nine (2–23). The *h*-index of retrieved articles was 113, meaning that at 113 documents of retrieved articles were cited at least 113 times, and this number might increase with time. Table 1 shows the annual number of publications from year 2000 to 2015 along with citation analysis for each year. As expected, the average number of citations per article was highest (48.6) for old articles, those published in year 2000, and was lowest (1.5) for new articles, those published in year 2015.

**Table 1:** Annual number of publications and citations on *Campylobacter* (2000–2015)

Year	Number of publications	% *N* = 5552	TC	C/A
2015	383	6.9	587	1.5
2014	448	8.1	1575	3.5
2013	435	7.8	29,19	6.7
2012	386	7.0	3700	9.6
2011	408	7.3	4978	12.2
2010	403	7.3	5639	14.0
2009	392	7.1	6476	16.5
2008	359	6.5	6826	19.0
2007	384	6.9	9346	24.3
2006	351	6.3	8276	23.6
2005	338	6.1	9912	29.3
2004	287	5.2	9582	33.4
2003	295	5.3	10,525	35.7
2002	239	4.3	9780	40.9
2001	256	4.6	10,630	41.5
2000	188	3.4	9138	48.6

*TC* total citations, *C/A* number of citations per article calculated by dividing the total number of citations retrieved for each year by the total number of publications for that year

### Subject areas

Retrieved documents were published in journals that belong to different subject areas. The majority (2783, 50.1%) was published in journals under the subject area of “immunology and microbiology”. Other common subject areas encountered were “medicine” (2471, 44.5%), “agricultural and biological science” (1625, 29.3%), and “biochemistry, genetics, and molecular biology” (1496, 26.9%). It should be noted here that there is an overlap in subject areas. For example, a certain specific journal could fit in more than two categories such as medicine and immunology/microbiology. Therefore, the sum of the percentage of all subject areas exceeded 100%. Table 2 shows the top ten subject areas of retrieved articles. The subject area “engineering” represents articles published mainly in the journal of *International Journal of Food Microbiology*. The subject area “chemistry” represents articles published in diverse journals that discuss chemical aspects of campylobacter microorganism.

**Table 2:** Top ten subject areas of publications on campylobacter (2000–2015)

SCR^a^	Subject area	Frequency	% *N* = 5552
1st	Immunology and microbiology	2783	50.1
2nd	Medicine	2471	44.5
3rd	Agricultural and biological sciences	1624	29.3
4th	Biochemistry, genetics and molecular biology	1496	26.9
5th	Veterinary	667	12.0
6th	Environmental science	437	7.9
7th	Pharmacology, toxicology, and pharmaceutics	185	3.3
8th	Engineering	130	2.3
9th	Chemistry	102	1.8
10th	Neuroscience	83	1.5

Equal countries were given the same ranking number, and then a gap is left in the ranking numbers

^a^ *SCR* standard competition ranking

### Most productive (active) countries

Countries that were most productive (active) in publishing documents on *campylobacter* were listed in Table 3. The USA was the most productive country (1309, 23.6%) followed by UK (829, 14.9%) and Canada (472; 8.5%). However, Netherlands ranked first (34.3) followed by UK (32.7) and Denmark (32.2) when countries were ranked based on the mean number of citations per article. Six countries in the top ten productive countries were in Europe. A total of 352 (6.3%) articles were authored or co-authored by all African and Middle Eastern countries. France had the lowest percentage (33.5%) of articles with international authors while Netherlands (57.7%) had the highest percentage of articles with international authors (Table 3).

**Table 3:** Top ten productive (active) countries in publishing articles on *Campylobacter* (2000–2015)

SCR	Country	Frequency	% *N* = 5552	TC	C/A	SCP	Percent	MCP	Percent
1st	USA	1309	23.6	36,321	27.7	847	64.7	462	35.3
2nd	UK	829	14.9	27,082	32.7	468	56.5	361	43.5
3rd	Canada	472	8.5	12,526	26.5	252	53.4	220	46.6
4th	Japan	310	5.6	4508	14.5	178	57.4	132	42.6
5th	Germany	275	5.0	4606	16.7	165	60.0	110	40.0
6th	Denmark	258	4.6	8315	32.2	143	55.4	115	44.6
7th	Netherlands	239	4.3	8199	34.3	101	42.3	138	57.7
8th	France	218	3.9	4433	20.3	145	66.5	73	33.5
9th	Australia	197	3.5	3524	17.9	108	54.8	89	45.2
10th	New Zealand	164	3.0	3051	18.6	88	53.7	76	46.3

*SCR* standard competition ranking, *TC* total citations, *C/A* number of citations per article calculated by dividing the total number of citations retrieved for each country by the total number of publications for that country, *h-index* Hirsch index, *SCP* single country publication (intra-country collaboration), *MCP* multiple country publications (inter-country publications)

### Most productive (active) institutions/organizations, authors, and journals

The most productive institution was the Technical University of Denmark (150; 2.7%) followed by the USDA ARS Russell Research Center RRC (USA) (2.5%) and Wageningen University and Research Centre (Netherlands) (2.0%). Three institutions in top ten productive institutions are located in the USA, two are in Netherlands, and two in the UK (Table 4). The Belfast Health and Social Care Trust is neither a university nor a research center yet ranked fourth with 108 publications. Analysis of these publications showed that 87 (80.5%) were collaborative articles between scientists in Belfast Health and Social Care Trust with researchers in universities and other research centers.

**Table 4:** Top ten productive (active) institutions/organizations in publishing articles on *Campylobacter* (2000–2015)

SCR^a^	Institution/organization	Frequency	% *N* = 5552	Country
1st	Danmarks Tekniske Universitet	150	2.7	Denmark
2nd	USDA ARS Russell Research Center RRC	138	2.5	USA
3rd	Wageningen University and Research Centre	110	2.0	Netherlands
4th	Belfast Health and Social Care Trust	108	1.9	UK
5th	Helsingin Yliopisto	98	1.8	Finland
6th	Utrecht University	93	1.7	Netherlands
7th	Iowa State University	91	1.6	USA
7th	Conseil national de recherches Canada	91	1.6	Canada
9th	Veterinary Laboratories Agency	90	1.6	UK
10th	USDA Agricultural Research Service, Washington DC	87	1.6	USA

Equal institutions were given the same ranking number and then a gap is left in the ranking numbers.

^a^ *SCR* standard competition ranking

The top ten productive authors are shown in Table 5. Four authors in the top ten productive list are from the UK, three from the USA, one is affiliated with WHO center, one from Japan, and one from Finland. The top ten productive journals in publishing articles on *Campylobacter* is shown in Table 6. The most active journal was the *Applied and Environmental Microbiology* followed by the *Journal of Food Protection* with 246 (4.4%) articles for each (Table 6). All journals in the top ten list had an impact factor. The range of IF for journals in top active list was from 1.67 to 3.99. All journals in the top ten list, with the exception of *Plos One*, are in specific field of microbiology, infection, food, poultry, or immunology. Three journals (*Plos One*, *Infection and Immunity*, and *Foodborne Pathogens and Disease*) in the top ten list follow an open access policy.

**Table 5:** Top ten productive (active) authors in publishing articles on *Campylobacter* (2000–2015)

SCR^a^	Author	Frequency	% *N* = 5552	Country
1st	Moore, J.E.	111	2.0	UK
2nd	Hänninen, M.L.	80	1.4	Finland
3rd	Wagenaar, J.A.	76	1.4	WHO
4th	Matsuda, M.	73	1.3	Japan
5th	Zhang, Q.	63	1.1	USA
6th	Millar, B.C.	57	1.0	UK
6th	Guerry, P.	56	1.0	USA
8th	Newell, D.G.	56	1.0	UK
8th	Wren, B.W.	56	1.0	UK
10th	Berrang, M.E.	49	0.9	USA

^a^ *SCR* Standard competition ranking. Equal authors were given the same ranking number, and then a gap is left in the ranking numbers

**Table 6:** Top ten productive (active) journals in publishing articles on *Campylobacter* (2000 – 2015)

SCR	Journal	Frequency	% *N* = 5552	IF
1st	*Applied and Environmental Microbiology*	246	4.4	3.668
1st	*Journal of Food Protection*	246	4.4	1.849
3rd	*Journal of Clinical Microbiology*	185	3.3	3.993
4th	*International Journal of Food Microbiology*	147	2.6	3.082
5th	*Journal of Applied Microbiology*	132	2.4	2.479
6th	*Epidemiology and Infection*	123	2.2	2.515
7th	*Plos One*	122	2.2	3.234
8th	*Poultry Science*	116	2.1	1.672
9th	*Infection and Immunity*	105	1.9	3.731
10th	*Foodborne Pathogens and Disease*	103	1.9	1.905

*SCR* standard competition ranking, *IF* impact factor

### Top cited articles

The top cited articles on *Campylobacter* [54–63] are shown in Table 7. The top cited articles were mostly in the field of molecular biology, pathophysiology, and public health. The highest number of citations attained was 1203 for an article on the molecular biology published in *Nature*. The top ten cited articles included seven research articles and three review articles.

**Table 7:** Top ten cited articles on *Campylobacter* (2000–2015)

Title	Year	Journal	Number of citations	Type of document
The genome sequence of the food-borne pathogen *Campylobacter jejuni* reveals hypervariable sequences [61]	2000	*Nature*	1223	Article
Increased rectal mucosal enteroendocrine cells, T lymphocytes, and increased gut permeability following acute *Campylobacter enteritis* and in post-dysenteric irritable bowel syndrome [62]	2000	*Gut*	776	Article
*Campylobacter jejuni* infections: update on emerging issues and trends [54]	2001	*Clinical Infectious Diseases*	597	Review
Bactericidal activities of plant essential oils and some of their isolated constituents against *Campylobacter jejuni*, *Escherichia coli*, *Listeria monocytogenes*, and *Salmonella enterica* [59]	2002	*Journal of Food Protection*	567	Article
Multilocus sequence typing system for *Campylobacter jejuni* [55]	2001	*Journal of Clinical Microbiology*	472	Article
Quinolone and macrolide resistance in *Campylobacter jejuni* and *C. coli*: resistance mechanisms and trends in human isolates [56]	2001	*Emerging Infectious Diseases*	426	Review
*N*-linked glycosylation in *Campylobacter jejuni* and its functional transfer into *E. coli* [63]	2002	*Science*	416	Article
Campylobacters as zoonotic pathogens: a food production perspective [4]	2007	*International Journal of Food Microbiology*	341	Review
Major structural differences and novel potential virulence mechanisms from the genomes of multiple campylobacter species [57]	2005	*PLoS Biology*	319	Article
Risk factors for sporadic Campylobacter infection in the United States: a case-control study in FoodNet sites [58]	2004	*Clinical Infectious Diseases*	302	Article

### Resistance profile

**Fig. 2:**
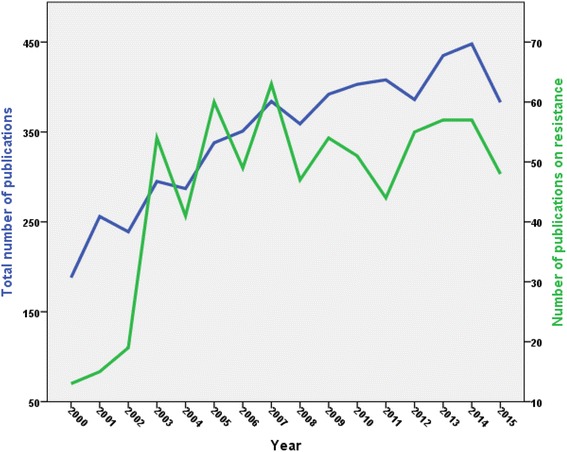
Annual growth of publications on Campylobacter and campylobacter-related drug resistance (2000–2015)

Analysis of retrieved documents for articles pertaining to *Campylobacter* drug resistance resulted in 728 articles, i.e., 13.16% of the total retrieved articles. The total number of citations received by articles on resistance was 14,191, a mean ± SD of 19.5 ± 30.2 citations per article and a median (IQR) of 10 (3–24). The *h*-index of retrieved articles on resistance was 53. Annual number of publications on drug-resistant *Campylobacter* showed a dramatic increase starting from year 2002 followed by a fluctuating plateau (Fig. 2). The dramatic increase in number of publications observed in 2002 was associated with 11 articles on the emergence of quinolone/fluoroquinolone resistance in *Campylobacter*. Top productive countries on *Campylobacter* drug resistance are shown in Table 8. Countries such as China (seventh) and Ireland (tenth) were among the top ten productive countries. The Iowa State University (38, 5.2%) was the most productive institution. The top ten cited articles [56, 64–72] are shown in Table 9. Four articles discussed on resistance to flouroquinolones. The article that received the highest citations was a review article published in the journal *Emerging Infectious Diseases* and titled *Quinolone and macrolide resistance in Campylobacter jejuni and C. coli: Resistance mechanisms and trends in human isolates*.

**Table 8:** Top ten productive (active) countries in publishing articles on campylobacter-related drug resistance (2000–2015)

SCR^a^	Country	Frequency	% *N* = 5552
1st	USA	168	23.1
2nd	UK	59	8.1
3rd	Canada	54	7.4
4th	France	38	5.2
5th	Japan	35	4.8
6th	Finland	28	3.8
7th	China	27	3.7
8th	Thailand	25	3.4
9th	Denmark	24	3.3
9th	Ireland	24	3.3
9th	Poland	24	3.3

Equal countries were given the same ranking number, and then a gap is left in the ranking numbers.

^a^ *SCR* standard competition ranking

**Table 9:** Top cited articles on campylobacter-related drug resistance (2000–2015)

Title	Year	Source title	Number of citations	Document type
Quinolone and macrolide resistance in *Campylobacter jejuni* and *C. coli*: resistance mechanisms and trends in human isolates [56]	2001	*Emerging Infectious Diseases*	426	Review
Antibiotic resistance in Campylobacter strains isolated from animals, foods, and humans in Spain in 1997–1998 [72]	2000	*Antimicrobial Agents and Chemotherapy*	218	Article
Fluoroquinolone-resistant Campylobacter species and the withdrawal of fluoroquinolones from use in poultry: a public health success story [71]	2007	*Clinical Infectious Diseases*	171	Review
Antimicrobial resistance among Campylobacter strains, United States, 1997–2001 [69]	2004	*Emerging Infectious Diseases*	166	Article
Enhanced in vivo fitness of fluoroquinolone-resistant Campylobacter jejuni in the absence of antibiotic selection pressure [70]	2005	*Proceedings of the National Academy of Sciences*	162	Article
Prevalence and antimicrobial susceptibility of thermophilic campylobacter in organic and conventional broiler flocks [65]	2001	*Letters in Applied Microbiology*	157	Article
Occurrence and resistance to antibiotics of *Campylobacter jejuni* and *Campylobacter coli* in animals and meat in northeastern Italy [67]	2003	*International Journal of Food Microbiology*	145	Article
In vivo selection of Campylobacter isolates with high levels of fluoroquinolone resistance associated with gyrA mutations and the function of the CmeABC efflux pump [66]	2003	*Antimicrobial Agents and Chemotherapy*	138	Article
Critical role of multidrug efflux pump CmeABC in bile resistance and in vivo colonization of Campylobacter jejuni [68]	2003	*Infection and Immunity*	132	Article
Antimicrobial susceptibilities of Campylobacter strains isolated from food animals in Belgium [64]	2001	*Journal of Antimicrobial Chemotherapy*	132	Article

## Discussion

In this study, a bibliometric overview of campylobacter-related publications was sought and presented. Our study showed a gradual and fluctuating increase in the number of publications with time. Most publications originated from developed countries. Campylobacter-related drug resistance publications also showed a noticeable increase in the past decade with quinolone and flouroquinolone resistance being most commonly emphasized. The result that the USA was the most productive was not surprising given that the USA ranked first in many other medical fields in quantity of publications [73, 74]. However, the USA ranked fourth in number of citations per article. One potential explanation for this discrepancy is the extent of international collaboration in publications from the USA compared to that of from the UK or the Netherlands. This study is the first bibliometric study on a zoonotic foodborne disease. No doubt that diarrhea associated with bacterial infections such as campylobacteriosis, particularly in developing regions, requires better monitoring and screening for types of bacteria involved and their susceptibility to traditional antibiotic therapy. More research efforts are required in this field.

The top ten cited articles on *Campylobacter* reveals that molecular biology and genomics of *Campylobacter* pathogen were the most important ones. Understanding the molecular biology and genomics will help understanding the pathogenesis of campylobacteriosis [75–78]. One of the top cited articles was about search for new bactericidal agents from plant active oils [59]. Search for inexpensive, available, and efficacious therapeutic agents for gastroenteritis is common in developing world where traditional herbal medicine is popular [79–81]. Other articles in the top ten list were on public health and the impact of campylobacteriosis on human health. Both food safety and antimicrobial resistance are core subjects of public health. Many reports have been published on foodborne infections and gastroenteritis in the form of outbreaks in Europe and many other countries. Similarly, combating antimicrobial resistance of campylobacter both at veterinary and human levels is a public health major concern [71, 82–85].

There are several studies regarding research activity in the field of infectious diseases in general and those pertaining to campylobacteriosis in specific. A review article on control measures of campylobacter-contaminated poultry meat indicated that current intervention measures are ineffective and there is urgent need for more fundamental research on *Campylobacter* [86]. A commentary that appeared in *Lancet* indicated that research on neglected diseases such as diarrhea is needed and a faster action is required [87]. The slow and fluctuating increase in research activity on campylobacter could be attributed to lack of funding to such globally common bacterial diseases in favor of other diseases such as HIV, tuberculosis, lymphatic filariasis, schistosomiasis, salmonellosis, and malaria [88, 89]. A study showed that 37% of infectious diseases affecting livestock animals are bacterial diseases and this should encourage funding agencies to support research on bacterial infectious diseases of livestock that could affect humans such as campylobacteriosis [90]. Unfortunately, the escalating funding for infectious diseases such as HIV created a shortage of funding for other diseases of global burden such as campylobacteriosis [91]. A study that compared research output and citations among three infectious diseases indicated that funding has a positive influence on research output and citations for a particular disease [92]. It could be concluded that in the case presented in this study for campylobacter research output that funding is an important factor, funding needs to be consistent and well planned based on global need, and collaboration in such funding is of extreme importance to help eradicate some of the neglected diseases of poverty such as diarrhea.

Our study showed that campylobacter-related drug resistance publications were reported from different parts of the world suggesting that such a problem is a global problem [93–98]. Antimicrobial resistance among *Campylobacter* has developed mainly as a result of irrational use of antimicrobial agents in veterinary practices and food industry [99, 100]. Resistance to macrolides, flouroquinolones, and beta-lactams has been reported [56, 101–104]. The mechanisms involved in resistance to flouroquinolone include mutations in the target enzyme called gyrase which is involved in bacterial DNA replication and repair [66, 103, 105]. Another important mechanism of resistance to quinolones includes efflux mechanism [68].

Our study has few limitations that need to be considered when interpreting the results. Like all bibliometric studies [38, 39, 42, 106–109], false positive and false negative results are unavoidable because there is no perfect and comprehensive research query. Therefore, we might have missed some articles either due to search query or to the use of title search rather than title/abstract search. Despite the fact that Scopus is one of the largest databases, there are some journals that are not indexed in Scopus, and therefore, articles on *Campylobacter* published in these unindexed journals were definitely missed. Furthermore, the citation analysis did not exclude self-citation, and therefore, ranking based on citations or citation per article might be misleading in certain cases.

## Conclusions

The number of publications on *Campylobacter* and on campylobacter-related drug resistance showed a clear rise in the past decade. Northern America and European countries are leading research on *Campylobacter*. Publications focused mainly on molecular biology/genetics and public health burden of campylobacteriosis. Research on rational drug use of antimicrobials in human, poultry, and animals is highly recommended to limit the emergence of antimicrobial resistance in *Campylobacter*. Furthermore, international collaboration is highly required particularly in implementing new diagnostic screening technologies for *Campylobacter* detection to ensure food safety.

## Abbreviations

CDC: Centers for Disease Control and Prevention
IF: Impact factor
SCP: Single country publications
SCR: Standard competition ranking
WHO: World Health Organization
